# Level of patients' satisfaction toward National Health Insurance in Istanbul City-Turkey

**DOI:** 10.1186/1471-2458-12-S2-A5

**Published:** 2012-11-27

**Authors:** Saad Ahmed Ali Jadoo, Sharifa Ezat Wan Puteh, Zafar Ahmed, Ammar Jawdat

**Affiliations:** 1 Department of Community Health, Universiti Kebangsaan Malaysia Medical Centre, Jalan Yaacob Latiff, 56000 Kuala Lumpur, Malaysia; 2 International Training Centre for Case mix and Clinical Coding, Universiti Kebangsaan Malaysia Medical Centre, Jalan Yaacob Latiff, 56000 Kuala Lumpur, Malaysia

## Background

Patients’ satisfaction is an indirect indicator of patients acceptance towards health management by healthcare providers. Healthcare reform of any magnitude needs to determine their external clients’ feedbacks measured through healthcare satisfaction. This study aimed to determine the level of patients’ satisfaction and its influencing factors toward the newly reformed national health insurance in Istanbul city, Turkey.

## Materials and methods

A cross sectional study was carried out in July-October 2011. A total of 345 heads of households have been selected by using simple random sampling selection method. Data were collected via household’s structured questionnaire and Patients' Satisfaction Questionnaire version II. The response rate was (89%) and data were analysed by using SPSS version 16.0.

## Results

Age of respondents was around 20 to 70 years old with the mean age of 41.97 ± 13.87 years. Majority of clients' had received tertiary education (54.5%) and most of them were currently employed (87.2%). Among the respondents, more than half were satisfied toward national health insurance (53.3%). Respondents were satisfied with domains of access to care (66.7%); availability of resources (52.2%); technical quality (56.5%); overall satisfaction (55.9%); continuity of care (68.7%) and humaneness (53.6%). In bivariate chi-square analysis, eight factors were shown to be significantly associated with level of satisfaction i.e. age, gender, marital status, education, occupation, self-perceived health status, area of residence and type of household’s plan. Further analysis, by using multiple logistic regression showed that these eight factors were also significant predictors of satisfaction level.

## Conclusion

This study showed that most of the respondents were satisfied with the existing national health insurance. Higher patients’ satisfaction was associated with improved access to care and continuity of care. However, light must be shed on availability of resources, technical quality and humaneness to improve overall patients’ satisfaction.

